# Promoting sustainable responses to the US opioid epidemic with community-academic partnerships: qualitative outcomes from a statewide program

**DOI:** 10.1186/s13011-022-00454-6

**Published:** 2022-04-07

**Authors:** David L.Driscoll, Alison Evans Cuellar, Vinod Agarwal, Debra Jones, Mary Beth Dunkenberger, Kathy Hosig

**Affiliations:** 1 University of Virginia School of Medicine, Charlottesville, USA; 2 Health Administration and Policy, George Mason University, Fairfax, USA; 3 Dragas Center for Economic Analysis and Policy, Old Dominion University, Norfolk, USA; 4 Human Health, Virginia State University, Petersburg, USA; 5 Virginia Tech, Blacksburg, USA; 6 Institute for Policy and Governance, Virginia Tech, Blacksburg, USA; 7 Population Health Sciences, Virginia Tech, Blacksburg, USA

**Keywords:** State Opioid Response, Community-academic partnerships, Program evaluation

## Abstract

**Background:**

Drug overdose deaths in the United States have continued to increase at an alarming rate. The Substance Abuse and Mental Health Services Administration (SAMHSA) distributed more than $7 billion between January 2016 and June 2020 to address the drug overdose crisis. The funds support evidence-based responses, including medications for opioid use disorder, and other prevention, treatment and recovery activities. Although the State Opioid Response (SOR) grants finance much-needed community level interventions, many of the services they support may not be sustainable, without ongoing assessment, evaluation and planning for continuation.

**Methods:**

This paper describes a statewide effort to support local entities through SAMHSA’s SOR grants in Virginia. Community agencies across the state participated in detailed needs assessment exercises with VHEOC investigators, and developed requests for proposals (RFPs) to sustain their SOR programs. The RFPs were then distributed to prospective academic partners at the five VHEOC universities, based on the required subject matter expertise identified in the RFP. All responsive proposals were then provided to the local agencies who selected the proposal most likely to meet their needs. VHEOC investigators also conducted an inductive, three-phase content analysis approach to examine the RFPs submitted to the VHEOC to identify nominal categories of support requested of the VHEOC investigators.

**Results:**

VHEOC Investigators received and coded 27 RFPs from ten community agencies representing four of five regions of the state. We identified six nominal categories of academic assistance with high inter-coder agreement. The six categories of support requested of the academic partners were program development and support, literature review and best practices, outreach and education, data analysis and interpretation, program evaluation, and grant writing assistance. Several RFPs requested up to three categories of support in a single project.

**Conclusions:**

Our analysis of the requests received by the consortium identified several categories of academic support for SOR-grantees addressing the drug overdose crisis. The most common requests related to development and maintenance of supportive collaborations, which existing research has demonstrated is necessary for the long-term sustainability of SOR-funded services. In this way, the academic partners reinforced sustainable SOR-funded programs. As the state opioid response program is implemented nationally, we hope that other states will consider similar models in response to the opioid crisis.

## Background

Drug overdose deaths in the United States have continued to increase at an alarming rate. In the 12-month period ending in January 2021, a record 94,134 Americans died from drug overdoses [1]. These deaths represent a 30.9% increase over the prior twelve months, and a nearly five-fold increase over the prior decade. Although few individuals who die of drug overdoses are using just one substance, the listed cause of overdose deaths are most often synthetic opioid analgesics (primarily fentanyl), followed by psychostimulants with abuse potential (primarily methamphetamines) [2]. The rate of overdose deaths appears to be escalating as the COVID-19 pandemic intensifies the social determinants of this disease of despair [3, 4].

The federal government has allocated substantial funding to respond to the drug overdose crisis. A popular provision of the 21st Century Cures Act, passed in 2016, authorized the Substance Abuse and Mental Health Services Administration (SAMHSA) to distribute $1 billion for programs that address opioid addiction and overdose [5]. Since that time, SAMHSA has continued to fund such programs through the State Opioid Response (SOR) grant program through which an additional $6 billion have been allocated to the states to provide evidence-based opioid use disorder prevention, treatment, and recovery services [6]. One key objective of these grants is to increase access to medications for opioid use disorder (MOUD) as part of a safe, effective, and clinically-appropriate comprehensive therapeutic approach [7]. In 2020, the SOR grants were further expected to address stimulant misuse and use disorders, including for cocaine and methamphetamine [8].

Although the SOR grants fund much-needed community level interventions, many of the services they support may not be sustainable [9]. One challenge faced by local grantees is that sustainable substance use prevention and treatment services rely on networks of collaborators, including payor sources for services. Although complex, these collaborative networks permit SOR-funded entities to overcome inter- and intra-organizational barriers, including limited fiscal resources and expertise, to sustain grant-funded programs [10]. Overcoming these barriers requires that grantees identify, leverage, and report on external factors, including partners and processes that will support new services throughout the service implementation pathway [9–12]. In addition to health service providers, these partners can include religious institutions, civic organizations, and law enforcement agencies who do not share a common or comprehensive understanding of the evidence base related to SUD treatment. There is a need for research to support the sustainable implementation of SOR-funded services. Community-academic partnerships (CAPs), such as the VHEOC, can both facilitate and conduct this needed research through a range of activities that may include needs assessments, program evaluation and translation of evidence-based practices [13]. Our approach is innovative because it describes a process by which researchers can establish CAPs rooted in the stated needs of community groups that provide proximal and distal benefits for community partners [14].

## Methods

This paper describes a model by which local colleges and universities can promote and bolster sustainable SOR-funded opioid response programs at the local level. We briefly describe the model by which the academic institutions involved in this project engaged with local and regional recipients of SOR-funds, and then detail the categories of support requested of the academic partner(s) in those collaborations. Our goal is to inform the development of similar academic-community collaborations to promote the sustainability of SOR-funded services and programs.

### Setting and design

This qualitative study evaluated the process and outcomes of a collaborative statewide consortium of public universities working with SOR-grantees across Virginia. These grantees, called Community Services Boards (or CSBs), are the local points of entry into specialty mental health, substance use disorder, and developmental services. The Virginia Department of Behavioral Health and Developmental Services (DBHDS) distributes SOR grant funding to the 39 CSBs across five regions of the state; Northern, Northwestern, Southwestern, Central, and Eastern. CSBs implementing or considering SOR applications were given the opportunity to apply for assistance from academic partners which could provide comprehensive support around prevention, treatment and data collection and analysis.

The academic consortium formed after several institutions independently convened community-academic workshops on the opioid overdose crisis at which CSB representatives advocated for a coordinated academic response to bolster substance use prevention, treatment, and management in their regions. The authors convened a five-university consortium to support the CSBs in implementing sustainable evidence-based services with funding from DBHDS. The university-based collaborative would become known as the Virginia Higher Education Opioid Consortium (VHEOC), leveraged academic expertise at George Mason University, Old Dominion University, University of Virginia, Virginia State University, and Virginia Tech.

The VHEOC governance committee, composed of the authors, conducted active outreach to Virginia’s CSBs from August 2019 through September 2020. The objectives of the outreach were to announce the availability of academic assistance for SOR-funded local services, provide a list of sample capabilities offered across the academic institutions, and to invite requests for proposals to support their services. The capabilities listed included, for example, technical support for prevention, treatment, and recovery programs, as well as the collection and analysis of program evaluation data. The mode(s) of outreach included email, telephone, and in-person site visits. In addition, representatives of the VHEOC presented at statewide behavioral health conferences attended by CSB leaders and other behavioral health providers, distributed brochures, both print and electronic, and maintained a project website with frequently asked questions and contact information for all participating institutions.

The specific modes of communication employed between VHEOC institutions and CSBs varied, but most culminated in meetings or workshops in which staff and leadership from one or more CSBs described their existing SOR-funded services and the capabilities necessary to improve or sustain them. The CSB leaders then participated in unstructured discussions with the VHEOC investigators to develop a request for proposals (RFP) using a template which explicitly linked funding to sustainability strategies. This template organized each RFP into five sections: nature of the problem to be addressed, purpose of the request to address the problem, primary point of contact for questions, date of completion, and outcomes or deliverables required.

The RFPs were reviewed by DBHDS for suitability for SOR funding, and then distributed by the VHEOC governance committee to faculty with relevant subject matter expertise across their respective institutions. Interested investigators, which could include VHEOC investigators, submitted proposals in response to specific RFPs through a VHEOC website. Faculty proposals were screened by a VHEOC review committee comprised of representatives of each partner university not including any submitting institutions. Those deemed responsive to the RFP were forwarded to the CSB for final review and selection. A VHEOC fiscal agent at the University of Virginia established the funding mechanism to support the academic partner(s) selected, managed the project in partnership with the local Principal Investigator(s) and the CSB(s). The PIs also provided quarterly progress reports to the VHEOC leadership to ensure that the funded project was conducted on time and within budget. The RFPs were also analyzed by the consortium leadership to identify common themes or categories of assistance requested of the academic partners.

### Data analysis

Six VHEOC investigators representing the five universities participated in an iterative, three-phase content analysis approach to examine the RFPs submitted by CSBs to identify nominal categories of support requested by CSBs [15]. In accordance with grounded theory analytic techniques [16, 17], the RFPs were initially coded a priori by at least one individual representative of each institution. This process resulted in an initial organizing framework of 18 prospective codes, which the team collectively applied to each RFP in a team-based deductive analytic process [15]. This approach allowed the team to combine and integrate predetermined codes for categories of support requested, and in some cases to identify emergent codes not captured in the initial codebook. These codes were entered into a revised codebook, which investigators from each institution employed to recode the RFPs individually. These results were integrated, intercoder was reliability determined [18], differences were discussed electronically, and the RFPs were recoded until the team achieved an acceptable intercoder reliability, resulting in six final codes.

## Results

The VHEOC received 27 requests for proposals between October 2019 and March 2020. The requests came from ten CSBs representing four of five regions of Virginia. Final coder agreement was 76% on six codes using Fleiss’s kappa, representing an acceptably high level of agreement [15]. The six categories of support identified by the VHEOC are described below in the order frequency requested. Several RFPs requested up to three categories of academic assistance.

### Program development and support

These requests sought assistance with the structure and/or process of SOR-sponsored programs. In many cases, these requests described a new service or program for which the academic partner could facilitate engagement with local partners and clients. One example was a request to support the development of a regional detox center. The academic partners were asked to provide research summaries on both the evidence base and business model case for supporting individuals from detox to recovery, and to establish research briefs to inform working group discussions of the new program, develop the program business model, and create the implementation checklist. This project also included a request for Outreach and Education to engage medical providers and consumers in the subsequent program.

### Literature review/best practices

These requests sought information on the state of the science to allow evidence-based development of new client services. One example was a project to conduct a literature review on best practices and toolkits for stigma reduction on behalf of a regional collaboration of CSBs. The academic team interviewed regional stakeholders to understand the local determinants of stigma related to treatment and recovery for SUDs, conducted a review of both the academic and gray literature related to stigma reduction, and as with the project above, developed public-facing messaging materials specific to the region to mitigate these determinants based on the evidence. This project exemplified many of the requests in this category because it was associated with others, particularly Outreach and Education.

### Outreach and education

These requests sought assistance in understanding barriers to, and provision of information to facilitate local support for, new SOR-sponsored programs. This included projects to develop and disseminate messages describing the safety and efficacy of MOUD as part of a safe, effective, and clinically-appropriate comprehensive therapeutic approach. The messages aimed to promote MOUD from a scientific perspective to assuage concerns and generate support for the program among key stakeholders, including those in local detention and drug court settings. The academic teams conducted formative research with, and developed messages tailored to, such program partners as health care providers and law enforcement personnel. This project exemplified many of the requests in this category because it involved the academic partner serving as a credible source of information on the evidence-based nature of the service being provided to skeptical stakeholders in the community.

### Program evaluation

These requests helped CSBs to document the impact of their SOR-supported services as required by SAMHSA for all SOR-sponsored services. One example was a project to analyze a 23-h Crisis Care Model. The academic team worked closely with the CSB leadership to develop data collection protocols, integrate their data management systems, identify process and outcome metrics related to the model, and develop data protocols by which the academic and CSB team collected and analyzed data from more than 3,000 patients in control and treatment groups. These data allowed the CSB to demonstrate to the sponsor and their program partners a significant reduction in repeated hospitalizations among service recipients.

### Data analysis/interpretation

These requests sought analytic support to answer operational questions related to specific SOR-sponsored services. One example was a project to develop a data dashboard system that allowed a regional drug prevention coalition to design, code, and share data related to their respective program outcomes, and empower collective planning and decision-making for SOR-related program support. The academic partners identified available sources of data and the indicators in those sources, assembled matrices by which the data are collected and managed, and provided training to CSBs to use the data dashboard.

### Grant writing assistance

These requests involved the collection and organization of data required for proposals to augment or continue SOR-related services. One example was a project to identify subpopulations with higher rates of morbidity and mortality associated with substance use or substance use disorder, and the social and environmental factors associated with those outcomes. The academic partners conducted a literature review and surveys of regional stakeholders to understand not only the disparities, but the local determinants of those disparities. These findings were used to develop a behavioral health disparities statement template that included the data sources and tools required to support the proposal, including references with links as appropriate.

## Discussion

Over the last five years, SAMHSA has allocated substantial SOR funding to state and local entities for services that prevent, treat, and manage recovery from SUD, including addiction to opioids. Existing research has demonstrated that the long-term sustainability of these programs likely depends on consortiums of community-based partners, including local colleges and universities, and local support for community-level prevention, treatment and recovery infrastructure. Our findings align with the recent literature, and have particular relevance to VHEOC investigators interested in strengthening the long-term sustainability of SOR-sponsored programs. Our project shows that a statewide consortium of academic partners can assist behavioral health agencies in determining what support they may require, and ensuring the provision of that assistance. This is a time-intensive process requiring development of trust between and among academic partners and community agencies through outreach, engagement, and technical assistance processes that are flexible to the preferences of community partners.

Our findings can serve as a starting point for this local engagement process by providing examples of six categories of support likely to be of assistance to community-based SOR grantees. We anticipate similar consortiums can refine and expand on these categories of academic support for sustainable SOR-sponsored services.

This study has several important limitations, the most substantial of which is the limited and self-selected nature of the study participants; CSBs providing requests for support. The consortium did not receive requests for assistance from every CSB in the state, and in some cases did not receive a response to repeated invitations to meet or discuss the VHEOC. While the requests came from CSBs representing four of five regions of Virginia, they may differ from other SOR-grantees in important ways with bearing on the categories of assistance likely to be requested from academic partners. Additionally, the qualitative data analyzed in this project may have been limited by the structured nature of the proposal template, which consisted of a series of questions focused on project objectives, timelines, and outcomes. VHEOC liaisons sought to overcome these constraints by eliciting open-ended and unstructured descriptions of SOR-funded services during the needs assessment process, and this process allowed for the inclusion of a variety of new and emergent objectives in the RFPs. Finally, the nature of the content analysis process is reductionist, simplifying complex requests and identifying boundaries between categories of requests that may not exist. For that reason, we include Table 1, which reveals which categories of requests most often co-occurred and thus may represent a shared set of needs on the part of the CSB leaders.

**Table 1:** Categories of Support by RFP

**RFP**	**Development and Support**	**Literature Reviews**	**Outreach/** **Education**	**Program evaluation**	**Data Analysis**	**Grant writing** **assistance**
1			X			X
2	X		X			
3						X
4	X			X		
5	X			X		
6	X	X		X		
7		X			X	
8	X	X			X	
9			X		X	
10				X		
11	X					
12		X		X		
13		X	X		X	
14		X	X	X		
15		X				X
16		X	X			
17	X		X			
18	X			X		
19	X	X		X		
20				X		
21		X	X			
22		X				
23	X	X				
24	X				X	
25	X		X		X	
26	X		X		X	
27					X	X
Total	13	12	10	9	8	4

## Conclusions

Our analysis of the requests received by the consortium identified several categories of academic support for SOR-grantees addressing the drug overdose crisis. Among the most common requests were assistance with the development and structure of opioid response programs, assessment of the evidence-base for those programs, and outreach and education to promote local acceptance and buy-in for them. These categories of academic assistance relate to development of supportive collaborations, which existing research has demonstrated is necessary for the long-term sustainability of SOR-funded services. Other categories of assistance were to collect, interpret, and evaluate the outcomes of SOR-supported services with the combined goals of demonstrating the effectiveness of new services to local partners and the sponsor, and to provide preliminary data to support new requests for funding. These categories of academic assistance were important for meeting the requirements of the SOR-grant mechanism, as well as any new mechanisms, with the objective of maintaining local partners while seeking new support for the SOR-sponsored services for individuals at risk of, or living with, SUD. In this way, the academic partners provide a unique source of assistance for SOR-funded programs at the local and regional level. As the state opioid response program is implemented nationally, we hope that other states will consider similar models in response to the opioid crisis.

## Data Availability

The needs assessments analyzed during the current study are not publicly available due to interviewee confidentiality. Though anonymized, information provided by participants may make them identifiable given the small sample pool. However, the resulting RFPs will be made available from the corresponding author on request.

## Abbreviations

CSB: Community Services Board
DBHDS: Department of Behavioral Health and Developmental Services
MOUD: Medications for Opioid Use Disorder
RFP: Request for Proposals
SAMHSA: Substance Abuse and Mental Health Services Administration
SOR: State Opioid Response grant program
SUD: Substance Use Disorder
VHEOC: Virginia Higher Education Opioid Consortium
