# Correction: Promoting sustainable responses to the US opioid epidemic with community-academic partnerships: qualitative outcomes from a statewide program

**DOI:** 10.1186/s13011-022-00477-z

**Published:** 2022-06-20

**Authors:** David L. Driscoll, Alison Evans Cuellar, Vinod Agarwal, Debra Jones, Mary Beth Dunkenberger, Kathy Hosig

**Affiliations:** 1 University of Virginia School of Medicine, Charlottesville, USA; 2 Health Administration and Policy, George Mason University, Fairfax, USA; 3 Dragas Center for Economic Analysis and Policy, Old Dominion University, Norfolk, USA; 4 Human Health, Virginia State University, Petersburg, USA; 5 Virginia Tech, Blacksburg, USA; 6 Institute for Policy and Governance, Virginia Tech, Blacksburg, USA; 7 Population Health Sciences, Virginia Tech, Blacksburg, USA

**Correction: Subst Abuse Treat Prev Policy 17, 26 (2022)**

**https://doi.org/10.1186/s13011-022-00454-6**

Following publication of the original article [1], we have been notified that one of the authors’ names was mentioned incorrectly. It should be as follows:

Given name – David L and family name – Driscoll.
